# Is tuberculosis crossing borders at the Eastern boundary of the European Union?

**DOI:** 10.1093/eurpub/ckt098

**Published:** 2013-06-27

**Authors:** Marieke J. van der Werf, Vahur Hollo, Teymur Noori

**Affiliations:** European Centre for Disease Prevention and Control (ECDC), Stockholm, Sweden

## Abstract

**Background:** The Eastern border of the European Union (EU) consists of 10 countries after the expansion of the EU in 2004 and 2007. These 10 countries border to the East to countries with high tuberculosis (TB) notification rates. We analyzed the notification data of Europe to quantify the impact of cross-border TB at the Eastern border of the EU. **Methods:** We used TB surveillance data of 2010 submitted by 53 European Region countries to the European Centre for Disease Prevention and Control and the World Health Organization Regional Office for Europe. Notified TB cases were stratified by origin of the case (national/foreign). We calculated the contribution of foreign to overall TB notification. **Results:** In the 10 EU countries located at the EU Eastern border, 618 notified TB cases (1.7% of all notified TB cases) were of foreign origin. Of those 618 TB cases, 173 (28.0%) were from countries bordering the EU to the East. More specifically, 90 (52.0%) were from Russia, 33 (19.1%) from Belarus, 33 (19.1%) from Ukraine, 13 (7.5%) from Moldova and 4 (2.3%) from Turkey. **Conclusions:** Currently, migrants contribute little to TB notifications in the 10 EU countries at the Eastern border of the EU, but changes in migration patterns may result in an increasing contribution. Therefore, EU countries at the Eastern border of the EU should strive to provide prompt diagnostic services and adequate treatment of migrants.

## Introduction

In 11 European Union and European Economic Area (EU/EEA) countries, >50% of the notified tuberculosis (TB) cases are diagnosed in individuals of foreign origin.^1^ In 2010, 21% of the notified multidrug-resistant tuberculosis (MDR-TB) cases in the EU countries were diagnosed in individuals of foreign origin.^1^ The number of notified TB cases in Norway and Sweden was 339 and 675, respectively, in 2010,^1^ of which >85% were TB cases reported to be of foreign origin. These figures show the importance of migration for TB control in the EU.

Since the collapse of the Soviet Union in 1992, there has been an explosive growth of TB in the 15 countries that were part of the Soviet Union.^2^^,^^3^. For example, the Russian Federation notified 34 TB cases per 100 000 population in 1990, and this tripled to 96 TB cases per 100 000 population in 2000.^4^ Similarly, MDR-TB has increased. Although high-quality longitudinal data are limited, data from Tomsk Oblast in Russia show a significant increase in the percentage of TB cases with MDR-TB, from 6.5% in 1998 to 13.7% in 2002.^5^

Since 2005, TB notifications have been decreasing in the World Health Organization European Region.^1^ However, countries in the east (non-EU countries) still have much higher notification rates than countries in the west (EU countries), 86.5 per 100 000 versus 14.6 per 100 000. Also, MDR-TB notification rates are higher, 7.1 per 100 000 in non-EU-countries as opposed to 0.3 per 100 000 in EU countries. Currently, 15 of the 27 MDR-TB high-burden countries in the world are located in the European Region6.^6^

After the expansion of the EU in 2004 and 2007, the Eastern border of the EU consists of 10 countries: Finland, Estonia, Latvia, Lithuania, Poland, Slovakia, Hungary, Romania, Bulgaria and Greece. These countries have a wide range in their percentage of foreign-born population, ranging from 0.8% of the total population in Romania to 16.4% in Estonia.^7^ In most of the EU countries at the Eastern border of the EU, the majority of the foreign-born population is reported to have been born in a non-EU country. In the four EU countries at the Eastern border for which information is available about the country of birth of persons born outside the country, a significant proportion of the foreign-born population residing in the country are from high-TB-incidence countries bordering the EU to the East (Russia, Ukraine or Belarus). For example, 29% of persons born outside Poland are from Ukraine and 11% from the Former Soviet Union, and in Latvia, 52% are from Russia and 13% from Ukraine.^8^ Given that TB notification rates are much higher in the countries bordering the EU to the East,^1^ this raises the question of what the impact is of cross-border migration in EU countries located at the Eastern border. Therefore, the objective of our analysis is to quantify cross-border TB at the Eastern border of the EU.

## Methods

The European Centre for Disease Prevention and Control (ECDC) and the World Health Organization Regional Office for Europe (WHO/Europe) collected TB surveillance data from the European Region countries for the year 2010.^1^ The data collection covers all 53 countries of the WHO European Region. EU/EEA Member States reported case-based data and non-EU/EEA countries reported aggregated data. Designated national surveillance institutions were responsible for reporting the data at the European level. Details about data collection methods can be obtained from the report *Tuberculosis surveillance and monitoring in Europe, 2012*.^1^

In EU/EEA countries, TB cases are defined according to agreed case definitions published by the European Commission (European Union Commission.2008/426/EC: commission Decision of 28 April 2008 amending Decision 2002/253/EC laying down case definitions for reporting communicable diseases to the Community network under Decision No 2119/98/EC of the European Parliament and of the Council [notified under document number C (2008) 1589]. OJ L 159, 18.06.2008, p. 46). Data from the non-EU/EEA countries of the European Region follow the WHO-recommended definitions. These define a ‘case of tuberculosis’ as a patient in whom TB has been confirmed by bacteriology or diagnosed by a clinician. A ‘definite case’ is defined as a patient with culture confirmation of *Mycobacterium tuberculosis* complex. In countries where culture is not routinely available, a patient with one sputum smear positive for acid-fast bacilli (AFB+) is also considered a definite case.

For the calculation of notification rates, country population denominators were obtained from Eurostat^8^ for the EU and EEA countries and from United Nations statistics^9^ for all others.

**Figure 1:**
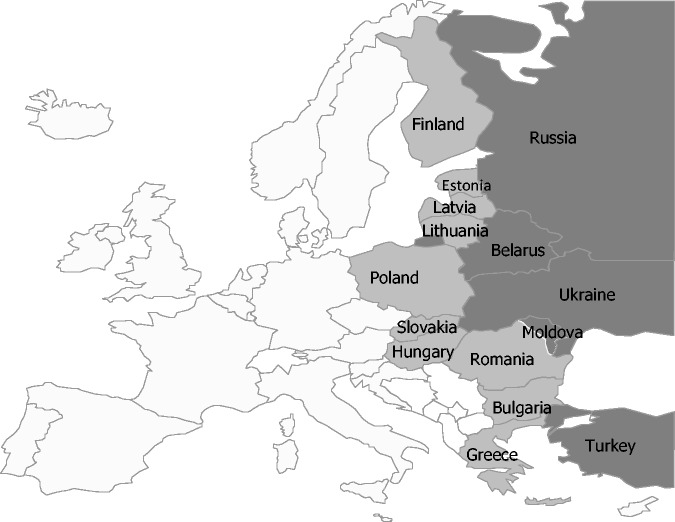
European Union and Economic Area (EU/EEA) countries and neighbouring countries. EU countries in light and middle grey, EU countries at the Eastern border of the EU in middle grey and non-EU countries bordering the EU to the East in dark grey

EU countries at the Eastern border of the EU are Finland, Estonia, Latvia, Lithuania, Poland, Slovakia, Hungary, Romania, Bulgaria and Greece (figure 1). For the purposes of this analysis, Cyprus was not considered an EU country at the Eastern border of the EU because the country does not have a land border with another country, and Norway was not considered a border country because it only borders to Russia in the far north of the country and migration using this border was considered to be minimal—also as the three main countries of birth in persons born outside Norway were Poland, Sweden and Germany.^10^

Notified TB cases were stratified by information on origin of the case (national/foreign). The geographical origin of TB cases was classified according to place of birth (born in the country/foreign born) or, if unavailable, citizenship (national/non-national). Finland, Estonia, Latvia, Lithuania, Slovakia, Romania, Bulgaria and Greece provided information on origin by place of birth. Data on citizenship were provided by Hungary and Poland. Cases with place of birth in an unspecified country of the Former Soviet Union were not taken into account for the detailed analysis (this applied to five cases notified in Finland). TB cases reported to have been born in the country or with citizenship of the country were categorized as ‘national’. Those born outside the country and without citizenship of the country were considered ‘foreign’.

Using information from Eurostat about the number of foreign-born in a selected number of countries,^10^ and the estimated TB incidence rate in 2010,^1^ we calculated the number of TB cases that are expected in individuals who migrated to EU countries at the Eastern border and were born in countries bordering the EU to the East.

Drug resistance surveillance methods varied across countries. All 10 countries had national coverage of drug susceptibility testing and provided drug susceptibility data to the ECDC/WHO-Europe database.^1^ In 8 of the 10 countries, >50% of the TB cases were culture positive, and also in 8 countries, DST results were available from >75% of the culture-positive TB cases. MDR is defined as resistance to at least isoniazid and rifampicin.

## Results

Of the 73 996 notified TB cases in the EU in 2010, 50.6% (37 433) were reported in the 10 EU countries located at the EU Eastern border. The notification rates ranged from 4.3 per 100 000 in Greece to 98.2 per 100 000 in Romania (table 1).

**Table 1:** Total TB case notification numbers and rates per 100 000 population and TB case notification numbers in foreign in countries located at the EU Eastern border, 2010

Country	Total number of notified TB cases	TB notification rate per 100 000	Number of notified TB in foreign^a^	Percentage foreign
Bulgaria	2649	35.0	2	0.1
Estonia	329	24.5	58	17.6
Finland	327	6.1	105	32.1
Greece	489	4.3	231	47.2
Hungary	1741	17.4	21	1.2
Latvia	934	41.5	62	6.6
Lithuania	1938	58.2	47	2.4
Poland	7509	19.7	46	0.6
Romania	21 078	98.2	38	0.2
Slovakia	439	8.1	8	1.8
Total	37 433	35.2	618	1.7

a: Data on citizenship were provided by Hungary and Poland, all other countries provided data on place of birth.

In the 10 EU countries located at the EU Eastern border, 1.7% of all notified TB cases (n = 618) were reported to be of foreign origin (table 1). Of these 618 TB patients, 104 (16.8%) were from other EU/EEA countries, 229 (37.1%) from non-EU countries in the European Region, 137 (22.2%) from Asia, 118 (19.1%) from Africa, 3 (0.5%) from North and South America and the country of origin was unknown for 27 (4.4%) (table 2). The two EU countries that never belonged to the Soviet Union, Finland and Greece, reported the highest percentage of foreign-born TB cases, 32.1% and 47.2%, respectively, and Bulgaria the lowest, 0.1%.

**Table 2:** Origin of foreign TB cases notified in EU countries at Eastern border of the EU, 2010

Country	Total number of TB cases of foreign origin	By region						
		EU/EEA^a^	Other European Region^b^	Rest of Asia^c^	Africa	Americas	Australia	Unknown
Bulgaria	2	0	2	0	0	0	0	0
Estonia	58	7	50	1	0	0	0	0
Finland	105	7	9	31	55	0	0	3
Greece	231	40	37	83	52	0	0	19
Hungary	21	15	3	2	1	0	0	0
Latvia	62	4	57	1	0	0	0	0
Lithuania	47	5	41	1	0	0	0	0
Poland	46	5	23	11	2	0	0	5
Romania	38	16	7	5	7	3	0	0
Slovakia	8	5	0	2	1	0	0	0
Total	618	104	229	137	118	3	0	27

a: European Union and European Economic Area.

b: 24 countries from WHO European Region, not belonging to EU/EEA.

c: Asia without countries from the European Region.

In total, only 173 (0.5% of the total number of notified TB cases) TB cases in EU countries located at the EU Eastern border were from neighbouring countries, i.e. countries physically bordering the EU to the East. Of those, 90 (52.0%) were from Russia, 33 (19.1%) from Belarus, 33 (19.1%) from Ukraine, 13 (7.5%) from Moldova and 4 (2.3%) from Turkey. The number of TB cases that were born or had citizenship in a country that is a direct neighbour to the East was limited. In Finland, 3 TB cases were from Russia; in Estonia, 34 were from Russia; in Latvia, 35 were from Russia and Belarus; in Lithuania, 15 were from Belarus; in Poland, 8 were from Belarus and Ukraine; in Hungary, one was from Ukraine; in Romania, six were from Moldova and in Greece, three were from Turkey. In Slovakia and Bulgaria, none of the TB cases originated from a direct neighbour country to the East.

Using information on the number of foreign-born individuals from Belarus, Russia, Ukraine and the Former Soviet Union that are living in Bulgaria, Latvia, Poland and Finland and the estimated TB incidence rate in Belarus, Russia, Ukraine and the Former Soviet Union, the expected number of TB cases in individuals from Belarus, Russia, Ukraine and the Former Soviet Union ranged between 6 and 181 (table 3). The notified number was 60–100% lower than the expected number of TB cases.

**Table 3:** Expected number of TB cases and notified number of TB cases among persons born outside the host country

Host country	Born in	Persons born outside the host country, 2011^a^	Estimated TB incident rate 2010 (per 100 000)	Expected number of TB cases	Notified number of cases in 2010
Bulgaria	Russia	18 700	104.9	20	0
	Ukraine	5900	101.2	6	0
Latvia	Russia	172 300	104.9	181	25
	Belarus	57 900	70.9	41	10
	Ukraine	42 400	101.2	43	17
Poland	Ukraine	155 500	101.2	157	6
	Former Soviet Union	59 200	105.8^b^	63	10
Finland	Former Soviet Union	48 700	105.8^b^	52	3

a: Data from reference (9).

b: Average estimated incidence rate of all Former Soviet Union countries.

In the 10 EU countries at the EU Eastern border, 1076 MDR-TB cases were notified; 29 (2.7%) of these MDR-TB cases were foreign, and 23 (79.3%) were from countries bordering the EU to the East.

Five countries bordering the EU to the East (Belarus, Moldova, Russia, Turkey and Ukraine) notified 226 514 TB cases in 2010^1^ (table 4). The notification rates ranged from 22.7 per 100 000 population in Turkey to 152.5 per 100 000 population in Moldova. In total, 20 631 MDR-TB cases were identified.

**Table 4:** TB case notifications and MDR-TB cases notifications in the five countries bordering the European Union to the East, 2010

Country	Number of notified TB cases	TB notification rate (per 100 000 population)	Number of cases tested for isoniazid and rifampicin resistance	Number of MDR-TB cases	MDR-TB (%)
Belarus	5554	57.9	3783	1576	41.7
Moldova	5447	152.5	2521	1082	42.9
Russia	162 553	113.7	49 267	12 387	25.1
Turkey	16 551	22.7	4957	250	5.0
Ukraine	36 409	80.1	14 034	5336	38.0
Total	226 514		74 562	20 631	27.7

## Discussion

There is little evidence to support the hypothesis that the higher TB rates in the former republics of the Soviet Union contribute to higher notification rates in the 10 EU countries at the Eastern border of the EU. Nevertheless, it is reasonable to expect that the higher notification rates in these neighbouring countries will contribute to rising TB rates in EU countries at the Eastern border of the EU, especially if migration patterns change.

In most EU countries at the Eastern border of the EU, the majority of the foreign-born population is born in or has citizenship from a non-EU country and often a country with a relatively high burden of TB.^4^^,^^10^ Although being foreign-born does not give an indication of recent migration and thus of importation of TB, it does give an indication of whether the person is at higher risk of developing TB.^11^

We found that the notified number of TB cases in EU countries at the Eastern border of the EU that can be attributed to citizens from neighbouring non-EU countries at the Eastern border of the EU was much lower than expected (table 3). This cannot be explained by incompleteness of reporting of the place of birth or citizenship of the TB cases, as the data were 98.5% complete for Finland and 100% complete for the other three countries. We used data from Eurostat to calculate the expected number of TB cases born in non-EU countries at the Eastern border of the EU now residing in EU countries at the Eastern border of the EU. If the number of migrants from non-EU countries at the Eastern border of the EU is actually lower than reported, i.e. because the EU countries at the Eastern border of the EU are transit countries and migrants do not remain in the country, our calculation would provide an overestimate of expected TB cases. An explanation for the low number of foreign TB cases can be that foreign-born individuals have insufficient access to health care facilities in their host countries and are therefore not diagnosed and notified. Migrants may also fear losing their jobs or being deported from the host country as a consequence of accessing the health care system and/or being diagnosed with a communicable disease. Another explanation is that mainly healthy individuals migrate and therefore persons born in another country are healthier compared with individuals who did not migrate from the country, i.e. a phenomenon known as the ‘healthy migrant effect’.^12^ Alternatively, the large difference between the expected number of TB cases and the notified number of TB cases can be due to a quick change of the disease pattern in the migrant population from a disease pattern that reflects the disease pattern in the country of origin to a disease pattern that reflects the pattern in the host country. This has been shown for non-communicable diseases.^13^

Several EU countries at the Eastern border of the EU, especially Finland, Greece, Hungary, Slovakia and Poland, have much lower TB notification rates (<20 per 100 000 population) compared with non-EU countries bordering the EU to the East.^1^ Therefore, one could assume that increasing migration flows from the TB-high-incidence countries bordering the EU can potentially give rise to increasing numbers of TB notifications in those EU countries receiving large amounts of migrants from these countries. An analysis of available data of migration flows to 8 of the 10 EU countries located at the EU Eastern border does not show any clear increasing or decreasing trend (table 5). Given the many factors that influence migration patterns, it is difficult to predict future migration trends and thus their effect on TB notification.

**Table 5:** Inflows of nationalities from countries bordering the EU to the East as a % of total inflows of foreigners, 2010 [OECD (2012) International Migration Outlook: SOPEMI 2012, OECD Publishing. http://dx.doi.org/10.1787/migr_outlook-2012-en]

Country	Annual average inflow of total inflow (%)	Annual average inflow of total inflow (%)
**Bulgaria**	**2006-2008**	**2009**
Turkey	14.1	18.7
Russian Federation	7.6	10.2
Moldova	6.3	3.4
Ukraine	5.7	4.9
**Estonia**	**2004–2008**	**2009**
Russian Federation	22.7	23.7
Ukraine	–	9.9
**Finland**	**2000–2009**	**2010**
Russian Federation	17.0	12.6
**Hungary**	**2000–2009**	**2010**
Ukraine	12	6.8
**Lithuania**	**2000–2009**	**2010**
Belarus	18.9	24.0
Russian Federation	23.3	23.4
Ukraine	13.7	13.7
Turkey	2.4	2.1
Moldova	2.6	1.5
**Poland**	**2000–2009**	**2010**
Ukraine	25	24.1
Belarus	7	7.1
Russian Federation	5.3	3.9
Turkey	1.8	2.7
**Romania**	**2005–2009**	**2010**
Moldova	41.9	28
Turkey	4.5	5.6
**Slovak Republic**	**2003–2009**	**2010**
Ukraine	9.7	10.6
Russian Federation	2.6	4

A much debated public health activity to reduce TB in migrants is the introduction of screening of migrants for TB before or just after entry. A systematic review reported a median yield of screening for TB disease of 0.18% in 14 national representative studies from EU countries.^14^ In these studies, three different screening strategies were used: (i) screening at port of entry, (ii) screening just after arrival in reception/holding centres and (iii) screening in the community after arrival in EU countries. None of the three strategies appeared to have a significantly higher yield. Another review concluded that screening of migrants at entry is not a very cost-effective TB control strategy.^15^

Alternatively, migrants can be screened for latent tuberculosis infection. This could be done by tuberculin skin testing, interferon gamma release assays or a combination of the two. Screening for latent tuberculosis infection may be cost-effective for migrants from countries with a high incidence of TB.^16^^,^^17^ However, adherence to chemoprophylaxis is often suboptimal.^18^

A survey in 2003 showed that none of the four EU countries at the Eastern border of the EU that participated (Romania, Hungary, Poland and Bulgaria) carried out TB screening for new entrant groups.^19^ Most migrants with TB will have TB when entering a country or develop TB in the first years after migration due to reactivation of a TB infection contracted before migration.^20^^,^^21^ Screening at entry and during the first years after migration might detect those cases early. Alternatively, TB in migrants can be diagnosed early if migrants have good access to free TB diagnosis and treatment.^22^^,^^23^

Migrants, but also nationals, diagnosed with TB might travel to other countries before or after TB diagnosis. Recently, a coordinated public health mechanism to guarantee TB prevention, diagnosis, treatment and care across borders has been proposed.^23^ The recommended minimum package provides minimum standards covering several areas: political commitment, financial mechanisms and adequate health service delivery (prevention, infection control, contact management, diagnosis and treatment and psychosocial support).

Our analysis used surveillance data that are routinely reported by countries to ECDC and WHO/Europe. The quality and comparability of reported data have improved considerably in recent years; however, the quality is known to vary between countries,^1^ Therefore, direct comparisons of the data presented across countries should be interpreted with caution.

We used two different variables to categorize TB cases into national or foreign, this might have resulted in allocation bias. To assess this, we analyzed the data of the five EU countries at the Eastern border of the EU that provided data on both place of birth and citizenship. In two countries, Greece and Slovakia, using place of birth or citizenship resulted in the same number of TB cases categorized as foreign. In three countries, using citizenship to categorize foreign provided less TB patients categorized as foreign; in Bulgaria, the two cases with place of birth outside Bulgaria had Bulgarian citizenship; in Lithuania, 43 of the 47 TB cases categorized as foreign using place of birth had Lithuanian citizenship and in Romania, 22 of 38 TB cases categorized as foreign using place of birth had Romanian citizenship. Therefore, using citizenship to categorize TB cases as foreign in Hungary and Poland might have resulted in an underestimation of the number of TB cases with foreign origin.

The ECDC and WHO/Europe TB surveillance database contains information about place of birth or citizenship. It does not have information on time since migration of the TB case or molecular typing information of the isolated strain. This makes it impossible to distinguish cases that were imported from cases that are due to transmission in the host country. ECDC is establishing a database with molecular typing information of MDR-TB strains identified in EU countries. This may facilitate monitoring and evaluation of recent transmission of MDR-TB in the EU versus importation from outside of the EU. The EU TB surveillance database^24^ can easily be adjusted to collect information about time since migration. However, as most EU countries are not collecting this information in their national TB surveillance databases, it will require a joint effort of the EU countries to make this information available at the EU level.

As of 2011, there are approximately 1 million ethnic Russians in the Baltic States [Latvia 556 422 (http://www.csb.gov.lv/en/notikumi/key-provisional-results-population-and-housing-census-2011-33306.html, accessed 10/12/2012.), Estonia 341 450 (http://www.stat.ee/34278, accessed 10/12/2012.) and Lithuania 174 900 (http://db1.stat.gov.lt/statbank/default.asp?w=1280, accessed 10/12/2012)]. Most of these Russians are migrants from the Soviet era and their descendants.^25^ Thus, most foreign TB cases diagnosed in Estonia, Latvia and Lithuania with place of birth as Russia are not recent migrants but have lived for a long time in the country. Thus, depending on the time frame used to define cross-border TB, our analysis may have overestimated cross-border TB in Estonia, Latvia and Lithuania.

Currently, migrants contribute little to TB notifications in the EU countries at the Eastern border of the EU. Only a small percentage (1.7%) of the TB patients in EU countries at the Eastern border of the EU are of foreign origin and of those only 28% are from countries bordering the EU to the East. As the notification rates are much higher in the countries bordering the EU to the East, increasing migration from these countries to EU countries at the Eastern border of the EU may result in an increasing rate of TB notifications in countries bordering the EU to the East. To ensure prompt diagnosis and adequate treatment of these migrants to limit transmission, it is essential that they have good access to TB diagnosis and TB treatment services in EU countries at the Eastern border of the EU.
